# Help-seeking experiences and intimate partner support in vulvodynia: A qualitative exploration

**DOI:** 10.1177/17455057241241866

**Published:** 2024-03-30

**Authors:** Athina Zoi Lountzi, Hannah Durand

**Affiliations:** Division of Psychology, Faculty of Natural Sciences, University of Stirling, Stirling, UK

**Keywords:** Help-seeking behaviours, partner support, vulva pain, vulvodynia

## Abstract

**Background:**

Vulvodynia is a poorly understood chronic pain condition characterized by persistent and unexplained pain in the vulva. Given the intimate nature of the pain, partners may play an important role in promoting self-management and help-seeking behaviours among women with vulvodynia.

**Objectives:**

The current study aimed to explore the role of partner support in pain experiences and help-seeking behaviours among women with vulvodynia.

**Design:**

A qualitative interpretive design was used.

**Methods:**

Ten women with vulvodynia (*M* age = 37.9 years) were interviewed using a semi-structured non-directive topic guide. Data were analysed using reflexive thematic analysis.

**Results:**

Three themes around help-seeking experiences were constructed from the data: (1) ‘It’s Been a Battle’ – Failed by the Healthcare System; (2) ‘It’s Just the Vulva’ – Dismissed by Healthcare Professionals; and (3) ‘I Diagnosed Myself’ – The Patient Becomes the Expert. Participants described negative help-seeking experiences characterized by long delays to diagnosis, lack of awareness and understanding from healthcare professionals, minimization of symptoms, and having to advocate for and demand care. A further three themes pertaining to partner support were also developed: (1) ‘That Person to Listen to You’ – Source of Emotional Support; (2) ‘Why Don’t You Try This?’ – Finding Solutions Together; and (3) ‘He Forgets that it’s Still There’ – Vulvodynia is a Foreign Concept. Partners provided emotional support and showed empathy and understanding, and practical support by accompanying women to medical appointments and help with pain management. However, participants felt partners’ understanding of vulvodynia was limited and that this impacted their relationships.

**Conclusions:**

Findings highlight a lack of continuity of care and multidisciplinary approach to treatment, with help-seeking experiences being mainly negative in this sample. Increasing public awareness of vulvodynia and improving healthcare access is crucial to improving physical and psychological outcomes for this group. Partners can play an important role in supporting people with vulvodynia; however, other outlets of support should be further explored.

## Plain Language Summary

Understanding How Women with Vulvodynia Seek Help and Get Support from Their Partners

Vulvodynia is a condition where women experience persistent and unexplained pain in the vulva. This pain can be quite personal and difficult to deal with. In this study, we wanted to understand how partners of women with vulvodynia help them cope with the pain and seek medical help. We interviewed 10 women with vulvodynia about their experiences of accessing healthcare for their symptoms, and how their partners affected these experiences. Many women faced challenges when seeking medical help, like delays in getting a diagnosis, healthcare professionals not understanding their condition, and their symptoms being downplayed. Women often had to be their own experts and advocate for their care. Partners of these women provided emotional and practical support, like going with them to medical appointments and helping them to manage the pain. However, some women felt their partners did not fully understand vulvodynia, and their worries sometimes strained their relationships. In conclusion, the study showed that there is a need for better healthcare for women with vulvodynia, including more awareness and easier access to treatment. Partners can be supportive, but other forms of support, for example, from friends, family, and other people with experience of vulva pain, should also be explored.

## Introduction

Vulvodynia is defined as pain in the female genital area, the vulva, that occurs for at least 3 months without a clear identifiable or visible cause, which may have potential associated factors.^1^ Expert consensus regards vulvodynia as a cluster of symptoms and potentially overlapping disease processes;^1^ these symptoms include soreness, burning, and stinging in and around the vulva, which can be triggered by touch (provoked vestibulodynia) and/or spontaneously triggered (generalized/unprovoked vulvodynia).^2^ It is estimated that vulvodynia affects from 10% to 28% of reproductive-aged women.^3,4^ Its incidence is estimated at 4.2%, with greater incidence among younger women and those with pre-existing sleep, psychological, and comorbid pain disorders.^5^

Vulvodynia has several important physical, emotional, and social consequences. Particularly, it is associated with increased rates of anxiety, depression, sexual dissatisfaction, and reduced self-esteem.^6^ It has a significant negative impact on women’s ability to perform daily activities, such as walking, sitting, practicing sports, as well as wearing certain types of clothing. Given the localization of the pain, vulvodynia can result in dyspareunia (painful sexual intercourse), which can have a negative impact on intimate relationships, as well as body image and mental health.^7,8^ Current treatments for vulvodynia include physiotherapy (e.g., pelvic floor exercises), muscle relaxants, nerve blocks, anti-depressants, anti-epileptic medication, cognitive behavioural therapy, and surgery.^9–12^ Treatment outcomes show low success rates, highlighting the need for further research as well as the adoption of a multidisciplinary, multimodal, and biopsychosocial approach to treatment.^13,14^ Self-management strategies (i.e., cognitive, behavioural, and physical techniques to address the physiological and emotional aspects of living with vulva pain) can help to relieve certain symptoms and prevent further irritation;^15^ however, the extent of use and effectiveness of self-management approaches is not known.

The aetiology of vulvodynia remains uncertain, but it is thought to be multifactorial.^13^ Research on why women experience vulvodynia has shown that psychosocial factors in conjunction with physical factors may influence the onset and continuation of pain, highlighting the need to adopt a biopsychosocial approach to understanding the condition.^16,17^ For instance, evidence suggests that vulvodynia may be associated with physical factors, such as genetics, hormones, inflammation, musculoskeletal problems, and neurologic mechanisms, as well as common comorbidities like interstitial cystitis.^1,9,18^ Vulvodynia has also been associated with anxiety and depression disorders, which increase the risk of onset as well as its persistence and level of pain interference, suggesting a potential bidirectional relationship.^19^ Adverse childhood experiences have also been associated with an increased risk of developing vulvodynia, with women with vulvodynia being more likely to have reported previous physical/sexual abuse.^20^ Nevertheless, the direction of causality is still unclear, and it is unlikely that the aetiology could be attributed to a single factor. It is considered that physical and psychosocial factors are intercorrelated causes of vulvodynia acting within a cyclical model.^17,21^

### Delayed diagnoses, help-seeking, and experienced injustice

Vulvodynia is largely underdiagnosed, with a diagnosis taking an average of 2 years and multiple visits to a variety of healthcare professionals.^22^ It is diagnosed through a process of exclusion, often leading to misdiagnoses of other conditions of the vulva, such as vaginismus, vaginitis, or pudendal neuralgia.^2,23^ Diagnosis is critical for any chronic pain condition as it can lead to feelings of validation, relief, as well as increased pain acceptance and commitment to manage the condition.^24^ Particularly for vulvodynia, receiving a diagnosis can aid women to articulate their symptoms and facilitate communication about their condition. The psychological consequences of vulvodynia on body image, self-esteem, depression, and anxiety can be negatively influenced by a lack of diagnosis, leading to women feeling abnormal, isolated, and embarrassed as well as underinformed about how to effectively manage their condition.^25^

Help-seeking rates among women with vulva pain are low due to myriad structural, social, and individual-level barriers. Approximately 50% of women with chronic vulva pain do not seek medical help, in large part due to perceived stigmatization from healthcare professionals.^26^ Women of colour, in particular, have reported elevated levels of medical mistrust and perceived discrimination, resulting in a tendency to avoid seeking assistance for vulvovaginal pain.^27^ Those who do seek care typically report seeing multiple different healthcare professionals from different specialisms (e.g., general practice, gynaecology, physiotherapy) in their pursuit of a diagnosis and treatment.^28^ Lack of consensus among healthcare professionals can impede confirmative diagnosis of vulvodynia, leading to further adverse psychological consequences.^29,30^ Psychological factors (e.g., anxiety, depression), negative self-perception and self-esteem, poor self-regulation skills (e.g., avoidance coping, catastrophic thinking), societal and internalized stigma, and interpersonal relationship factors can also inhibit help-seeking behaviour.^31^

A common theme reported in studies on vulvodynia is experienced injustice from the healthcare system and healthcare professionals.^32,33^ That is, women report feeling disbelieved and stigmatized by doctors, with their symptoms being minimized and misunderstood.^34,35^ In a previous study, women reported a lack of compassion and understanding from healthcare professionals about their symptoms.^30^ In addition, they described how difficult it had been to obtain a diagnosis, and how persistent they had to be in order to do so.^30^ The various challenges patients experience in seeking help for vulva pain can deter future help-seeking and exacerbate negative health- and wellbeing-related outcomes.^26,31,35^ However, it should be noted that research in this area to date is limited in its representativeness of lower socioeconomic status groups, disabled individuals, and minority gender, sexual, and racial and ethnic groups. Individuals from these groups may have more negative healthcare experiences and face additional and unique barriers to accessing care.^27^

### Partner role

Considering the nature of the pain in vulvodynia, the role of intimate partners should not be overlooked. Partners may trigger pain during sexual activities, but they also witness and have their own reactions to the pain.^36^ The emotional consequences of vulvodynia on women’s self-esteem and body image, as well as on their mental health may also affect their intimate relationships.^37^ Nevertheless, partners can offer a form of social support, which has been found to be crucial for people with chronic pain conditions.^38^

Current research on partner support has mainly focused on relationship satisfaction and the influence of partner responses to the pain. For instance, it has been found that men’s relationship satisfaction may undermine their motivation to understand women’s pain experiences, which could potentially lead to low partner support and result in increased pain catastrophising and pain intensity.^39^ Partner responses to women’s pain have been categorized as promoting an avoidant or an adaptive approach to the pain. Adaptive and facilitative partner responses have been associated with lower pain and disability as well as lower depression and anxiety compared with avoidant responses.^39^ Partner supportiveness was also associated with increased sex life-related satisfaction and decreased sex life-related distress in a recent study involving women of colour with chronic vulvovaginal pain.^40^ Consequently, intervention research on couples has emphasized the need to increase disclosure and empathetic response to women’s pain to ensure relationship satisfaction and effective coping with vulvodynia symptoms.^41^ Recent literature has hitherto failed to explore how partner support can influence other behaviours associated with the management of vulvodynia, such as seeking medical help, willingness to engage with treatments, as well as exploring alternative options. This literature is also limited by its near exclusive focus on young adults, which limits its generalizability to other age groups. Furthermore, extant qualitative research on relationships in the context of vulvodynia has predominantly involved couples being interviewed together, which may bias participants’ responses. Interviewing women with vulvodynia about their perceptions of partner support without their partner being present may facilitate more open expression.

### Current study

The aim of the present study was to explore the help-seeking behaviours of women with vulvodynia and the role of partner support in promoting help-seeking and self-management. Semi-structured online interviews were conducted to provide an in-depth understanding of women’s help-seeking experiences and the role of partner support in help-seeking behaviours and management of vulvodynia.

## Method

### Design

A qualitative interpretive design was used.

### Ethical considerations

Ethical approval was granted by the General University Ethics Panel at the University of Stirling. All participants gave written informed consent via an online form before being interviewed. Consent forms were stored securely and separately from the audio-recordings and transcriptions of interviews to protect participants’ anonymity. To protect the confidentiality of participants and comply with General Data Protection Regulation (GDPR), transcripts were anonymized, and data were stored on a secure online university-affiliated system to which only the research team had access.

### Participants

Women with vulvodynia were eligible to take part in the study. Participation was open to women who had self-diagnosed vulvodynia as well as those who were formally diagnosed by a healthcare professional. Inclusion criteria were being above the age of 18 years and currently or previously having been in an intimate relationship. The only exclusion criterion was not being able to complete the study in English.

A total of 10 participants took part in the study. Evaluation of data adequacy^42^ and information power^43^ was used to guide our decision to cease recruitment in light of time constraints and lack of funding. Participants were recruited via online social media platforms, including Facebook support groups for people with vulvodynia. Participants ranged in age from 25 to 57 years (*M* = 37.9, *SD* = 12.23 years). The median time elapsed between symptom onset and diagnosis was 3 years (interquartile range = 5.5). Table 1 provides participants’ demographic information.

**Table 1.** Participant characteristics.

Pseudonym	Age	Ethnicity	Age at symptom onset	Age at formal diagnosis
Louise	41	White	28	28
Jane	27	White	16	25
Lisa	57	White	50	Self-diagnosed
Emily	43	White	35	37
Christina	30	White	17	22
Mary	56	White	51	52
Anna	25	Hispanic	18	21
Rose	29	White	20	23
Ann	45	White	21	22
Bethany	26	White	16	24

### Procedure

A purposive convenience sampling approach was used wherein the study was advertised via social media platforms and the study information was shared by relevant professional networks and patient groups. This approach offered an efficient and cost-effective way to access the target population. Potential participants were asked to provide their email address via a secure online form if they wished to take part in the interview. Those who provided their email address (*N* = 24) received a detailed participant information sheet and were asked to sign a digital consent form if they wished to proceed with the study. Once consent was provided, an interview date was scheduled.

Ten women with vulvodynia gave consent and ultimately took part in the study. Semi-structured interviews were conducted online via Microsoft Teams using a non-directive topic guide. Interviews were conducted by the lead researcher (A.Z.L.). The guide was formed based on existing literature and reviewed by each member of the research team for suitability. A copy of the topic guide is available as supplemental material. Interviews lasted between 30 and 45 min. Participants were asked to provide a preferred pseudonym prior to the interview. The interviews were recorded to facilitate the data analysis. Interview recordings were transcribed verbatim for analysis and subsequently destroyed. Data collection took place over a 3-month period (May–July 2023). To aid comprehensive reporting, the Consolidated Criteria for Reporting Qualitative Research (COREQ)^44^ were followed. A COREQ checklist is provided as supplemental material.

### Data analysis

Reflexive thematic analysis (TA) according to Braun and Clarke^45,46^ was used to analyse the data. Reflexive TA was selected as it provided a flexible approach to identifying patterns of meaning to address the research question: ‘What are the help-seeking experiences of women with vulvodynia and what is the role of perceived partner support?’ The researcher’s epistemological position of critical realism aligned with the reflexive TA approach, as it recognizes the vital role of the researcher’s subjectivity during the process of analysis.

The six steps of reflexive TA were followed in an iterative manner. Following completion of the interviews, the lead researcher (A.Z.L.) familiarized themselves with the data by engaging with the transcripts multiple times. During this phase, the researcher asked critical self-reflective questions (e.g., what was guiding their interpretation of the data? How would they feel in the discussed situation?) as well as critically reflecting upon what might influence each participant’s interpretation of the situation they had experienced.^47^ Initial codes were constructed through an inductive and exploratory process. Codes were then grouped into clusters to generate initial themes, each organized around a central meaningful and distinct concept. The researcher named and defined each theme while also reviewing all interview excerpts to ensure the themes were appropriate. The research team met frequently during this process to discuss the data and analysis, and to collaboratively review codes, categories, and themes for comprehensiveness, coherence, and grounding in the data.

### Reflexivity

It is critical to highlight the researcher’s role in the process of conducting this research project as well as in the data analysis. The personal experiences of the researchers could have influenced the interpretation of, and the inferences made about the help-seeking behaviours of women as well as the role of partner support. Importantly, the lead researcher conducted both interviews and analysis of data, and thus, pre-existing perspectives could have influenced the reported observations. The researcher’s subjectivity is a crucial and integral component of reflexive TA and, thus, it can constitute a strength rather than a weakness by ensuring transparency and self-reflection.^46,48^

Several steps were taken to ensure that the study and analysis were conducted rigorously and principles of reflexivity were adhered to. Reflexive writing was used to record the researchers’ viewpoints and choices throughout the research process, generating a record that could be consulted later. Frequent meetings wherein the researchers identified and challenged assumptions and personal beliefs ensured that collaborative reflexivity was practised throughout the research process.

## Results

Results have been sub-divided into help-seeking experiences and partner support-related themes.

### Help-seeking experiences

Three themes were constructed from the data on help-seeking behaviours and experiences of women with vulvodynia. These themes are described with illustrative participant quotes, below.

#### Theme 1: ‘It’s Been a Battle’ – Failed by the Healthcare System

This theme describes the healthcare system-related issues that participants faced in seeking help for vulvodynia, from seeking a diagnosis to accessing treatment. Participants discussed how seeking help for their vulva pain felt like a constant battle. They felt that the responsibility for their care lay on their shoulders, and that they had to actively demand treatment and support from healthcare professionals. They described their determination to persevere in seeking help despite the challenges of this for their wellbeing:

Anna: My experience is that I have managed my own care. [. . .] It’s hard to advocate for myself at this point when I have been disregarded so many times.

Mary: GPs have been fairly negative. Not that they’ve been horrible, they just haven’t been helpful. [. . .] I mean, that day I got really upset and I just said, ‘look, you’re not listening to me. You don’t seem to understand. I’m in a huge amount of pain.’ If you’re asking me about my journey, it’s been a battle. I’ve managed it all myself.

Participants expressed their frustration at the long process of accessing a diagnosis and getting help for their pain. They felt that appropriate and comprehensive medical care was not readily available for women with vulvodynia in the United Kingdom:

Anna: I was seeking treatment, but no one knew really what was happening and everyone was like, ‘well, at this point, we’ve tried antibiotics and antifungals and nothing. So, it must be in your brain.’ [. . .] I shouldn’t have had to struggle nearly two years of not really knowing what that was and trying to find the right provider.

Ann: I just feel like, you know, I just kind of gave up because I feel like the health industry kind of failed me in that regard. Nobody was like, ‘let’s try things and see if you feel better.’ It was more like, ‘well, that’s just the way it is.’ And I kind of accepted that that was just the way it was.

Jane: I’m currently 27, and I would say I first developed vulvodynia about age 16, so it was quite a long time after my symptoms started [that I received care]. My actual pain between UTIs was quite ignored. [. . .] I’ve got called complicated and told to wait until I moved to a bigger town. When I went to university at the GP, I definitely felt like I was taken more seriously, but once they had ruled out the things that they were able to test for, I felt very much abandoned.

Having finally received their diagnosis, many participants felt they were not provided with the time and support that they needed to process information and ask questions during medical appointments. This resulted in further delays to accessing appropriate treatment:

Anna: I wasn’t given the space to really process what I was being told before asking me if I had any questions. Everything was so fast that I didn’t have a chance to really think about what had happened or what questions I could have possibly had. And then I left the office, and I had a million questions. So, I really felt like it was this pipeline of, like, ‘this is what we do, and you just get on with it and we’ll see you in three months or whenever I have available.’ Which was frustrating because I have already waited about four months to see you and I get ten minutes of care.

Participants also expressed uncertainty and confusion about where to go and what kinds of medical professionals to seek out for help with their vulva pain. This confusion was compounded by the lack of a clear referral pathway for people with vulva pain and extended to the vast variety of treatment options and self-management approaches, which participants felt they had to discover and disentangle for themselves:

Lisa: It’s not in the arena of healthcare specialists and consultants, so it’s still seeming a bit mysterious as to who you should actually go and see in terms of a medical professional. Just this whole confusion about what sort of health professional you should see, this comes up constantly on the Facebook pages. Every conversation has this sort of thread going through it or, ‘who have you seen? Have you gone to this? Have you tried this?’

Bethany: I feel like even the specialists don’t have all the information. Out there was good information. It’s kind of dispersed different places, it’s not like the information’s all in one place.

Although various treatment options for vulvodynia exist, participants struggled to access these. Many participants felt they had no choice but to seek private healthcare at personal financial cost to receive adequate care and find relief from their symptoms:

Lisa: I can’t get coverage for physiotherapy, for instance, which is very disappointing because I probably need ongoing physiotherapy [. . .], which at the moment I pay for privately.

Jane: I went to see a private physiotherapist because when I tried to see an NHS [National Health Service] physiotherapist, I was told there was no referral pathway for people like me.

Rose: Certain things cost so much, too, and that’s part of the reason as to why I try not to go as often as I probably should, just because it costs a lot to still have one appointment.

Fundamentally, participants felt that most healthcare professionals they had encountered were ill-equipped to deliver care and self-management guidance to people with vulvodynia. Those who eventually found a specialist or treatment approach that worked for them lamented the time and energy this had taken:

Rose: I was talking to my physical therapist when I went to pelvic floor therapy and she knew so many things on how to deal with it mentally and physically in different ways that my gynaecologist hadn’t even talked about it all, so it was like even the professionals, I feel, are not as versed on how to deal with that pain.

Ann: I even think throughout the years when I have gone to see a gynaecologist, they just don’t even know how to treat it. [. . .] I have a friend who does pelvic floor therapy, she’s the one who said to me, ‘hey, they have treatments now for that and you should really pursue it,’ and so I feel late to the game.

#### Theme 2: ‘It’s Just the Vulva’ – Dismissed by Healthcare Professionals

This theme reflects the overwhelming sense of dismissal that participants imparted during their interviews. Participants felt that their pain and other symptoms were minimized and downgraded by healthcare professionals, making them feel abandoned and dismissed. They described feeling as if they were expected to just accept it and learn to live with the pain:

Rose: I had a few doctors that would just tell me, ‘it’s all in your head.’ [. . .] I was almost in tears because I was in so much pain and he looked at me and, he laughed, and he was like, ‘you shouldn’t be in any pain at all.’

Emily: You are repeatedly told ‘I can’t see anything, it looks healthy. There’s nothing wrong,’ but you know there’s something wrong. [. . .] He was like, ‘well, I’m not going to prescribe it for you because you shouldn’t be on it. It’s a nothing condition.’

Participants highlighted the lack of public and healthcare professional awareness about vulvodynia and its impact on women’s physical and mental wellbeing as being detrimental to their care. They felt this lack of knowledge and insight into the lived experience of chronic vulva pain perpetuated stigma and acted as a key barrier to accessing adequate care and self-management support:

Jane: It felt very much like they didn’t know what to do [. . .] there wasn’t resources for it, and sometimes I just saw doctors who didn’t really seem to care or didn’t really seem to understand the level of pain, or how impactful it was. I’ve never actually spoken to another person with vulvodynia, that I know of, and so there has been that sort of isolation too.

Emily: No one understands, no one’s heard of it. So, you constantly have to explain what it is to people. In the medical profession, they should know. I don’t expect them to know everything, but they should know roughly what it is or roughly where it’s dealt with. [. . .] They just think, ‘Oh well, it’s just the vulva.’ But that little area creates a whole lot of pain.

#### Theme 3: ‘I Diagnosed Myself’ – The Patient Becomes the Expert

This theme illustrates how participants actively took charge of their healthcare through self-directed information-seeking, spurred by dissatisfaction with the ineffective care they had previously received. Participants engaged in extensive information-seeking behaviour, largely motivated by a lack of faith in healthcare professionals to deliver care:

Anna: At this point I know more about this condition than most doctors. I have read 90% of the papers that are out there about this condition.

Ann: I kind of gave up on saying, ‘hey, can you help me?’ because I didn’t think they would help me. I diagnosed myself and I basically went into the doctors [and said], ‘I think I have vulvodynia.’

Many described having to seek out information on their symptoms themselves in order to facilitate their formal diagnoses. Information-seeking prior to attending medical appointments was viewed by many as essential to support self-advocacy efforts and access specialist referral:

Jane: I quite forcefully went back to the doctors and said, ‘I know I’ve got all that, all my symptoms. Please let me see a specialist,’ and that’s when I got diagnosed.

### Partner support

Three themes were constructed from the data on partner support. These themes are described with illustrative participant quotes, below.

#### Theme 1: ‘That Person to Listen to You’ – Source of Emotional Support

Participants emphasized the importance of their partners in providing emotional support, particularly through attentive listening, patience, and understanding and validation of their pain experiences:

Louise: He was always very, very thoughtful. He was, you know, he would never push me into anything that I didn’t want to do. [. . .] There wasn’t much he could do really, other than offer emotional support and just, listen. I think that’s the main thing.

Mary: We know each other really well and we’ve been through a lot together, so he’s been very understanding, and he has been there when I’ve had moments, especially at the beginning when I just broke down and cried for like ten minutes at a time and he’d come and give me a cuddle.

Jane: That person to listen to you, to listen to all your theories and let you rant at them a little bit about your experiences and validates that it wasn’t okay and that it’s not fair.

While partners were described as emotionally supportive, some participants noted occasional lapses in attentiveness or understanding:

Rose: There are some times in my relationship where, you know, he’s very supportive, and there’s other times where it’s like, I feel like, you know, they don’t really listen, because it’s not something that they have to deal with, I guess. So, it’s almost like in one ear out the other.

Ann: I would say, you know, he felt bad for me, but at the same time, I think he was kind of like, ‘well, I don’t know what to do for you.’

Emily: I felt a little bit let down. I couldn’t talk to my partner about it and him understand that I was actually in pain, and I couldn’t cope. I think he just got fed up with me moaning about it.

#### Theme 2: ‘Why Don’t You Try This?’ – Finding Solutions Together

Participants recounted collaborative efforts with their partners to manage vulvodynia symptoms, including sharing pain experiences and exploring potential solutions together. Partners provided informational and instrumental support to aid pain self-management and healthcare-seeking:

Christina: It has really been just talking through things with my husband. He’s the one that suggested the heating pad. I am just like, ‘this is what it feels like,’ and one of us will say, ‘oh well if it feels kind of like this other pain that I’ve had before, this has helped me, so why don’t you try this?’ or something similar.

Emily: When you go to any appointment that you’re really anxious about, to have somebody there to support you, it’s a lot easier. [. . .] it’s just reassuring to know that he was outside and if I needed him, he could come in and help me if I needed it.

Broadly, participants were satisfied with the instrumental support they received from their partners; however, some expressed a preference or desire for more emotional support:

Anna: He will remind me of, like, ‘hey, have you done your physical therapy homework yet? Do you want me to do it with you? Do you want me to help with it?’ ‘Cause sometimes he can help with it. He’s a doer. He’s not the type of person that thinks a lot about how much talking about it might help or how much trying to get out of a certain headspace might help. He’s a ‘what can we do about it?’ person.

#### Theme 3: ‘He Forgets that it’s Still There’ – Vulvodynia is a Foreign Concept

Some participants reported that, although they received support from their partners to seek help and manage their symptoms, the consequences and impact of the pain had put a strain on the relationship. For many, this strain was attributed to their partner’s limited understanding of vulvodynia and its impacts, resulting in avoidance behaviour within the relationship. Some women felt their partners were fearful of provoking pain through sexual contact, while others felt their partners did not fully comprehend the nature or chronicity of their condition:

Anna: So, it’s that combination of, ‘how can I help you get rid of it?’ while also avoiding anything that might trigger it. [. . .] Even sitting down at home to have a meal, we don’t do that anymore. We are always in activities that will keep us distracted from engaging with one another, [. . .] we’re frozen in this state of fear.

Emily: He did go out of his way to try and help me. [. . .] It has been him who said, ‘we’ll get you private help,’ [. . .] but because the flares are less frequent, I think he forgets that it’s still there. I think that if he was here now and you asked him what I’ve got, he wouldn’t be able to tell you.

Rose: It’s almost like I wish that he could feel what I feel, because it’s like, ‘if you understood that level of pain, that intense level of pain, you would, like, you would be totally . . . you would [do things] so much differently.’

Issues around sex and intimacy were commonly discussed in the interviews. Many participants felt anger and sadness at the impacts of vulvodynia on their partners and their relationships. While some expressed a kind of mourning for the relationships that could have been, others described how their partners’ efforts to foster a shared healthy and satisfying sex life had brought them closer together:

Jane: It definitely puts a big strain on a relationship, but, at the same time, I guess it makes me think, if my partner can support me with this, ‘you found a really supportive, kind person who really loves you for you.’ It has maybe helped me develop a relationship with someone who truly cares about [me] and all of [my] difficulties.

Christina: My husband is very patient. He does his best. He’s been very great about making [sex] as enjoyable for me as possible. It is possible, so I think the fact that he’s seen it can be possible and enjoyable makes him more motivated. [. . .] It makes me feel closer to him because he’s trying so hard and actually listening, you know, and I know not everybody has that.

## Discussion

The current study aimed to explore the help-seeking behaviours and experiences of women with vulvodynia, with emphasis on the role of partners in supporting help-seeking and self-management. Participants described mostly negative experiences of help-seeking. Most experienced considerable delay in receiving a diagnosis after presenting with painful symptoms. Participants felt there was a lack of awareness about vulvodynia among healthcare professionals as well as a lack of referral pathways. Women reported visiting a variety of medical specialists in an attempt to receive a diagnosis and treatment. For many, even after receiving a diagnosis, suitable treatment and support was lacking. The experience of seeking help for vulvodynia was also emotionally challenging, leading to feelings of isolation, fear, hopelessness, and frustration.

Participants felt their partners were understanding when it came to their vulva pain, and that they provided emotional and practical support for pain management. Partners were perceived as attentive and concerned about the pain, and as helpful and supportive by the participants. Participants described how their partners flexibly took charge of more household responsibilities, encouraged women to rest, and assisted them in finding solutions to effectively manage their symptoms. However, for some, there was a sense that partners did not, or could not, fully understand their experience of vulvodynia.

### Help-seeking experiences

Various significant challenges associated with accessing a diagnosis, treatment, and self-management support for vulvodynia are reflected in the current study. Participants felt they had been failed by public healthcare systems due to a lack of clear information about where to seek help, as well as the lack of referral pathways. Many were also disappointed at the lack of knowledge among healthcare professionals about vulvodynia, particularly in primary care. Many participants ultimately sought private healthcare before receiving their diagnosis, which has significant cost implications. Accessing private healthcare led to more positive help-seeking experiences, as women felt their symptoms were better understood and investigated, and that they were offered adequate time to explore treatment options. These individuals felt they had no choice but to seek help privately; however, those without the financial resources to access private healthcare have no choice but to persevere with under-resourced public healthcare services that have hitherto failed to deliver appropriate care. While limited healthcare resources affect everyone, it is important to ensure that equal standards of care are accessible to all women experiencing vulva pain.^35^ These findings are not particularly surprising; indeed, many of the systemic issues identified in the current study were highlighted in a National Institute of Health report on vulvodynia in 2012.^49^ That little progress has been made to address these issues in the past decade is concerning and highlights the need for further investment in efforts to advance our scientific understanding of vulvodynia, to enhance its clinical care, and to improve outcomes for those who live with vulva pain.

In line with the extant literature, participants reported visiting multiple different healthcare professionals within the same field, and a median 3-year gap between symptom onset and obtaining a diagnosis.^13,14^ There was high variability in time to diagnosis in this sample, with one participant being self-diagnosed without formal confirmation from a healthcare professional despite presenting with symptoms for 7 years. There are several factors that may explain this variability, in particular, poor understanding among healthcare professionals of vulvodynia and its multifactorial aetiology.^50^ More research is needed to understand the aetiology of vulvodynia, while the implementation of more educational and professional development programmes for healthcare providers will improve vulvar care.

Participants perceived a lack of sensitivity and empathy from their healthcare providers, which can have significant detrimental effects on wellbeing and help-seeking behaviours.^30,51^ Many expressed disappointment and concern that their doctors viewed vulvodynia as a psychosomatic disorder. For example, one participant stated:

I’m not convinced that it’s a good idea to be saying that this stuff is psychological, because I don’t think it is. I think it’s good to have psychological support when you’re going through something like this, but I don’t think it’s good when medicine assumes that pain is caused by, like, you just not doing well in your head. I think that’s dangerous [. . .] ‘it’s all in your head,’ [. . .] that’s damaging to hear.

While psychosocial factors are implicated in vulvodynia and its diagnosis,^50^ there is strong evidence of physical components to this condition.^1^ Associations with psychosocial variables do not and should not invalidate patients’ pain experiences. There is a need to appropriately educate and inform healthcare professionals about the body–mind connection and its implications for chronic pain so women in their care are understood and appropriately supported. Given the complex interplay of physical and psychosocial factors that influence the presentation of vulvodynia,^13,14^ a multidisciplinary and multimodal approach to treatment is critical to ensure holistic care.

The interviews reflected the weight of responsibility participants felt to ensure they received appropriate care for their vulvodynia. Most participants had self-diagnosed vulvodynia before receiving a formal diagnosis, and ultimately felt that had they not investigated their symptoms themselves they would not have been formally diagnosed. They also felt they had to become strong advocates for themselves and demand care, which is consistent with the previous literature.^25,33^ Women’s pain is more poorly understood and undertreated compared with pain in men,^52^ and women are disproportionately required to self-advocate and convince others that their pain warrants care,^35,53^ particularly for female-specific conditions. This issue is systemic and constitutes a threat to human rights and public health.^54^ Data-driven system-wide changes to policy and practice are required to ensure equitable healthcare delivery and effective utilization of healthcare resources.

Participants in this study engaged extensively in health information-seeking behaviour, and highlighted challenges in identifying comprehensive and reliable resources on vulvodynia and its management. Health information-seeking behaviour is a common strategy to support adjustment to chronic illness and facilitate self-advocacy efforts;^55^ however, separating reliable health information from mis- and disinformation can be a challenge, particularly for those with poorer health literacy. A comprehensive and evidence-based vulva pain ‘toolkit’ disseminated via a reliable source such as the National Health Services (NHS) website could be a useful resource for individuals seeking healthcare and self-management support for vulva pain.

### Partner support

Research on partner support has shown that facilitative responses to pain are associated with lower pain and disability, compared with solicitous responses, which have been linked to greater pain through an increase in avoidance responses towards intercourse.^36,39^ In the current study, which focused on overall support and help-seeking, participants expressed that their partners mainly provided emotional support when it came to their vulvodynia. Some participants also highlighted that their partners helped them to identify practical solutions and accompanied them to medical appointments, which could be argued to increase motivation and courage to persevere in seeking help.^4,38^

In the current sample, low partner support, distracting, and negative responses to vulva pain, such as irritation and frustration, were seldom described. Indeed, it was the participants themselves who felt frustration and anger at the negative effects of vulvodynia on their intimate relationships. Some participants revealed that the negative effects on their intimate relationships motivated them to keep seeking help and finding solutions. Therefore, women’s emotional state and sense-making of the effects of vulvodynia should be taken into consideration when exploring partner roles in future studies.

Importantly, it was also mentioned that vulvodynia was a foreign concept to some partners, leading to increased fear and worry about causing pain, which can negatively impact relationships. As previously highlighted, solicitous responses to pain may lead to avoidance of intimacy^36,39^ and, hence, it could be suggested that supportive partners’ emotions and understanding of the condition also have a critical role in the pain experience. Nonetheless, women reported feeling lonely and isolated, irrespective of partner support. This was further highlighted by women expressing frustration at the lack of support from friends and family and poor public awareness of vulvodynia. While partners play an important role, having someone to speak to who experiences a similar situation or being able to communicate your pain to friends and family appears to be critically important to women. This is consistent with social support theory in the context of chronic illness;^56^ people with chronic conditions may seek support from a subset of their wider social network, or indeed seek out additional sources of support in the online environment. People with chronic conditions may find social support from others with shared experiences more beneficial than support from those who do not have the same condition.^57^ Many participants in this study sought out information and support via social media, particularly from dedicated online communities of people with vulvodynia. However, unmoderated online activity can lead to increased anxiety and frustration, which could be counterproductive.^58^ Understanding the social support needs of individuals with vulvodynia may enable the development of evidence-based peer support networks, which have been shown to be beneficial for women with other pain conditions.^59,60^

### Limitations and future recommendations

The current study provides important insights into the help-seeking experiences of women with vulvodynia and the role of partner support. Nonetheless, several limitations should be considered. Although the current sample was more widely representative in terms of age than those used in previous research on this topic, it consisted predominantly of white, well-educated, cisgender, heterosexual women who could access private healthcare and were willing to disclose personal information about their experiences and intimate relationships, which hinders the representativeness of the findings. This can be largely attributed to the convenience sampling approach. Due to resource limitations, social media was used to recruit participants. Recruitment via social media has well-established limitations in terms of bias and representation.^61^ That said, the cost-effectiveness, reach, and rapidity of targeted social media recruitment make it an important research tool, particularly for women’s health research, which has been historically underfunded.^62,63^ Future research should aim to understand the experiences of a greater diversity of individuals, including but not limited to those from ethnic and gender minority groups and people with disabilities. This could be achieved by diversifying recruitment approaches (e.g., clinic-based recruitment and community-based approaches, such as engagement with online support groups, advocacy groups, and relevant community groups and events).

Clinical characteristics of the sample should also be considered. To enhance inclusivity, given the barriers to diagnosis of vulvodynia,^64^ self-diagnosed women were eligible to participate. Ultimately only one self-diagnosed participant took part in this study; however, given that vulvodynia symptoms overlap with several other conditions (e.g., pudendal neuralgia), it is possible that some self-diagnoses of vulvodynia are not clinically accurate. The experiences of those who struggle to access a diagnosis warrant discussion within this literature; that said, researchers may need to consider whether inclusion of those without a formal diagnosis may be at odds with their research aims. In addition, we did not ask participants to report any comorbidities that may have contributed to their vulva pain (e.g., Ehlers-Danlos syndrome, mast cell activation syndrome, pelvic congestion syndrome, endometriosis, pudendal neuralgia, hip abnormalities, etc.) and therefore influenced their help-seeking experiences.

Finally, although the research was conducted in the United Kingdom, participants had accessed healthcare for vulvodynia in several different countries. This limits the scope of the recommendations for specific health services that can be made based on these data. Nevertheless, the consistency of participants’ experiences highlights that the issues described occur across healthcare settings and contexts. Future research should aim to identify patient-centred targets for improvement in vulvodynia care in key healthcare settings (e.g., primary care) to optimize patient outcomes. Healthcare professionals and policymakers’ perspectives should be included in this work to ensure that recommendations are feasible and implementable so that findings can be effectively translated into practice.

## Conclusion

Women with vulvodynia described negative help-seeking experiences, characterized by multiple visits to healthcare professionals and large gaps between symptom onset and diagnosis. The lack of a multidisciplinary approach to treatment and referral pathways to specialists exacerbated these negative experiences. The role of partners is crucial in providing emotional support and motivating women to seek help, find solutions, and manage their symptoms. Nevertheless, both women and their partners’ understanding of the condition should be considered. Accessible resources and guidelines for all women about where to seek help for vulva pain, what specialists to see, and what treatment options are available could be of benefit. More work is needed to improve education and public awareness about vulvodynia, which can in turn reduce stigma associated with the condition and improve health and wellbeing outcomes. Finally, research on other outlets of support should be expanded, focusing on the role of peer support and moderated online communities.

## Supplemental Material

sj-docx-1-whe-10.1177_17455057241241866 – Supplemental material for Help-seeking experiences and intimate partner support in vulvodynia: A qualitative exploration Supplemental material, sj-docx-1-whe-10.1177_17455057241241866 for Help-seeking experiences and intimate partner support in vulvodynia: A qualitative exploration by Athina Zoi Lountzi and Hannah Durand in Women’s Health

sj-docx-2-whe-10.1177_17455057241241866 – Supplemental material for Help-seeking experiences and intimate partner support in vulvodynia: A qualitative exploration Supplemental material, sj-docx-2-whe-10.1177_17455057241241866 for Help-seeking experiences and intimate partner support in vulvodynia: A qualitative exploration by Athina Zoi Lountzi and Hannah Durand in Women’s Health

## Declarations

**Ethics approval and consent to participate:** This study was approved by the General University Ethics Panel at the University of Stirling (Ref: GUEP 2023 13334 10027). Written informed consent was obtained from all participants via a secure digital form prior to study participation. All procedures were conducted in accordance with the ethical standards of the institutional research committee and with the 1964 Helsinki declaration and its later amendments or comparable ethical standards.

**Consent for publication:** Not applicable.

**Availability of data and materials:** Data supporting the present findings are not publicly accessible due to ethical responsibilities for data protection. Pseudonymized data, however, may be available on reasonable request to the corresponding author.
