# Supplementary material for: Help-seeking experiences and intimate partner support in vulvodynia: A qualitative exploration
Source: Womens Health (Lond). 2024 Mar 30;20:17455057241241866. doi: 10.1177/17455057241241866 (PMC10981854; doi:10.1177/17455057241241866)
Supplement: sj-docx-1-whe-10.1177_17455057241241866 – Supplemental material for Help-seeking experiences and intimate partner support in vulvodynia: A qualitative exploration [file sj-docx-1-whe-10.1177_17455057241241866.docx]

**Consolidated criteria for reporting qualitative studies (COREQ): 32-item checklist**

**Title:** Help-seeking experiences and intimate partner support in vulvodynia: A qualitative exploration

| **No. Item** | **Guide questions/description** | **Details** |
| --- | --- | --- |
| **Domain 1: Research team and reﬂexivity** | | |
| *Personal Characteristics* | | |
| 1. Inter viewer/facilitator | Which author/s conducted the interview or focus group? | AZL conducted the interviews. |
| 2. Credentials | What were the researcher’s credentials? E.g. PhD, MD | BSc, MSc |
| 3. Occupation | What was their occupation at the time of the study? | MSc Health Psychology student |
| 4. Gender | Was the researcher male or female? | Female |
| 5. Experience and training | What experience or training did the researcher have? | AZL received formal training in qualitative research methods as part of their MSc Health Psychology training and was supervised and supported in conducting the research by HD, an experienced qualitative health researcher. |
| *Relationship with participants* | | |
| 6. Relationship established | Was a relationship established prior to study commencement? | The researchers had no prior relationship with the participants. |
| 7. Participant knowledge of the interviewer | What did the participants know about the researcher? e.g. personal goals, reasons for doing the research | The participants were informed that the research would contribute towards the award of MSc Health Psychology for AZL. |
| 8. Interviewer characteristics | What characteristics were reported about the interviewer/facilitator? e.g. Bias, assumptions, reasons and interests in the research topic | The researcher was closely engaged in the research process and therefore unable to eliminate personal bias. |
| **Domain 2: study design** | | |
| *Theoretical framework* | | |
| 9. Methodological orientation and Theory | What methodological orientation was stated to underpin the study? e.g. grounded theory, discourse analysis, ethnography, phenomenology, content analysis. | Reflexive thematic analysis was used in this study. An inductive approach was adopted. |
| *Participant selection* | | |
| 10. Sampling | How were participants selected? e.g. purposive, convenience, consecutive, snowball | A purposive convenience sampling approach was used. |
| 11. Method of approach | How were participants approached? e.g. face-to-face, telephone, mail, email | Participants were invited to take part via social media posts and/or email newsletters from relevant organisations. |
| 12. Sample size | How many participants were in the study? | Ten participants were interviewed. |
| 13. Non-participation | How many people refused to participate or dropped out? Reasons? | Fourteen individuals who requested the study information ultimately did not consent to take part in the study. These individuals were not required to give a reason for non-participation. All participants who consented to take part in the research were interviewed. |
| *Setting* | | |
| 14. Setting of data collection | Where was the data collected? e.g. home, clinic, workplace | Interviews were conducted online via video-conferencing software. |
| 15. Presence of non-participants | Was anyone else present besides the participants and researchers? | Not applicable |
| 16. Description of sample | What are the important characteristics of the sample? e.g. demographic data, date | Participants were women with vulvodynia who had experience of seeking healthcare for their vulva pain and had been or were currently in an intimate relationship. |
| *Data collection* | | |
| 17. Interview guide | Were questions, prompts, guides provided by the authors? Was it pilot tested? | A semi-structured interview topic guide was used. This was developed based on existing literature and reviewed by all members of the research team. |
| 18. Repeat interviews | Were repeat interviews carried out? If yes, how many? | Not applicable |
| 19. Audio/visual recording | Did the research use audio or visual recording to collect the data? | Interviews were audio recorded to facilitate detailed transcription. |
| 20. Field notes | Were ﬁeld notes made during and/or after the interview or focus group? | AZL took notes on their impressions and observations both during and after interviews as needed. |
| 21. Duration | What was the duration of the interviews or focus group? | Interviews lasted between 30-45 minutes. |
| 22. Data saturation | Was data saturation discussed? | The concept of data saturation is not consistent with the reflexive thematic analysis approach per Braun and Clarke. Evaluation of data adequacy and information power was instead undertaken to ensure the dataset was suitably rich to address the research aim. |
| 23. Transcripts returned | Were transcripts returned to participants for comment and/or correction? | Transcripts were not returned to participants for comment or correction. |
| **Domain 3: analysis and ﬁndings** | | |
| *Data analysis* | | |
| 24. Number of data coders | How many data coders coded the data? | AZL coded the data. Codes were then reviewed by HD for comprehensiveness and coherence. |
| 25. Description of the coding tree | Did authors provide a description of the coding tree? | Open coding was initially performed. This consisted of transcripts being read and re-read thoroughly and descriptive codes being assigned to sections of text. The content of the transcripts was constantly compared with codes that had been previously established. After forming the codes, they were grouped into categories, which were then grouped into themes. |
| 26. Derivation of themes | Were themes identiﬁed in advance or derived from the data? | All themes were derived from the data. |
| 27. Software | What software, if applicable, was used to manage the data? | Interviews were transcribed in Microsoft Word. No other software was used to manage the data. |
| 28. Participant checking | Did participants provide feedback on the ﬁndings? | Participants did not provide feedback on the findings. |
| *Reporting* | | |
| 29. Quotations presented | Were participant quotations presented to illustrate the themes/ﬁndings? Was each quotation identiﬁed? e.g. participant number. | Participant quotations are presented to illustrate the findings. Each quotation is identified using the relevant participant’s chosen pseudonym. |
| 30. Data and ﬁndings consistent | Was there consistency between the data presented and the ﬁndings? | There is consistency between the data presented and the findings. |
| 31. Clarity of major themes | Were major themes clearly presented in the ﬁndings? | All major themes are clearly presented in the findings. |
| 32. Clarity of minor themes | Is there a description of diverse cases or discussion of minor themes? | Not applicable |
