# Supplementary material for: Help-seeking experiences and intimate partner support in vulvodynia: A qualitative exploration
Source: Womens Health (Lond). 2024 Mar 30;20:17455057241241866. doi: 10.1177/17455057241241866 (PMC10981854; doi:10.1177/17455057241241866)
Supplement: sj-docx-2-whe-10.1177_17455057241241866 – Supplemental material for Help-seeking experiences and intimate partner support in vulvodynia: A qualitative exploration [file sj-docx-2-whe-10.1177_17455057241241866.docx]

**Supplemental material**

**Interview Guide**

Thank participant for taking part in the study

- Introduce myself
- Reassure them about their anonymity
- Highlight that the interview is informal, there is no judgement, and they can share only what they feel comfortable with
- Express my interest in advancing the research on vulvodynia
- Explain the interview process, I am just interested in your experiences, opinions, and thoughts
- Inform participant that they can pause or end the interview at any time
- Do you have any questions before we begin?

**Interview Questions:**

1. Tell me about your experience of vulvodynia

- 1. *Prompts:*

- How does vulvodynia affect your life

2. How would you describe your experience of seeking help for your pain up to this point?

- 1. *Prompts:*

*-* How did you first seek help? What was that like?

3. Why do you think it was easy/challenging?

*3.1 Prompts:*

- Would you tell me more about it, perhaps using an example?
- How did you feel about this?
- Was this what you expected?

4. What prompted you to seek help?

5. Where did you seek help from? (Medical professional, friends/family, online communities?)

6. How would you describe your relationship with your partner when it comes to vulvodynia?
 *6.1 Prompts:*

- Did the pain begin prior to or during this relationship? How has it affected/changed your relationship?
- How do you feel about this?
- What were your expectations?

7. How has your partner supported you seeking help for vulvodynia?
(If not support from partner, did you seek support elsewhere, friends or family, and if so, how have they supported you?)

*7.1 Prompts:*

- How would you have liked to be treated by…?
- How did you feel about this?
- What would you have liked to have happened?
- Tell me more about your relationship with …

8. Is there anything else you would like to add regarding your experience of seeking help for vulvodynia?

**End of Interview**

- Thank you
- Do you have any questions?
- Explain what will happen next, referring back to the participant information sheet and debrief
- Thank you for your time
